# Monochromatic light measurement via geometric phase and Fourier-transform spectroscopy method

**DOI:** 10.1038/s41598-022-17211-1

**Published:** 2022-07-28

**Authors:** Florin Garoi, Ionut Nicolae, Petronela Prepelita

**Affiliations:** Laser Department, National Institute for Laser, Plasma and Radiation Physics, 409 Atomistilor Street, Magurele, 077125 Ilfov Romania

**Keywords:** Optical sensors, Optoelectronic devices and components, Imaging and sensing, Design, synthesis and processing, Imaging techniques

## Abstract

The paper introduces a method for the measurement of monochromatic light using the geometric phase shift in a polarizing interferometer and applying the Fourier transform spectroscopy method. This is achieved with no mirror displacement or change in the actual optical path difference. Our method uses the rotation of a half-wave plate with increments on the order of degrees of arc, which is easier to control and reliable to reproduce. This approach provides flexibility in choosing the spectral range and a novel way of performing such measurements. It is demonstrated how the achromatic nature of the geometric phase allows only for monochromatic measurements to be acquired. The method is described theoretically and its performance is validated via measurements of several low-coherence light sources. Three possible applications of the method are also described, two of which are advantaged by using a detector array and, surprisingly, the achromaticity of the geometric phase.

## Introduction

Fourier-transform spectroscopy (FTS)^1,2^ takes advantage of the Fourier mathematics and interferometry. It is a technique in which spectra are acquired from measurements of the coherence of a radiation source, be it temporal or spatial. Therefore, both time-domain^3–6^ and space-domain^7–10^ measurements are applicable to Fourier-transform spectroscopy. As the name suggests, FTS requires a Fourier-transform to turn the raw data into an actual spectrum and uses the interference phenomenon rather than dispersion. It usually implies an interferometer in which displacement along the optical axis of one of the mirrors introduces changes in the optical path difference (OPD) between the interfering waves. Consequently, a scanning of the interferogram of the incident radiation source is achieved; and by applying the Fourier transformation, its spectrum as a function of spatial frequency is recovered. Alternatively, if the interferogram is scanned at constant speed and data points are taken at constant time intervals, the interferogram is a function of time. In this situation, the Fourier transformation gives the spectrum as a function of temporal frequencies.

In this research we applied the FTS principle in a polarizing interferometer^11–13^ while keeping it to zero path difference (ZPD) and, hence, removing any translation of the mirror. With such an interferometer and a suitable polarization phase-shifter^14–16^ it is possible to use the Pancharatnam geometric phase^17–19^ to scan the interferogram. Thus, instead of concerning ourselves with controlling fine displacements of the mirror (~ tens of nanometers level), we need only to control rotations on the order of degrees of arc, for the same spectral range. However, this method only allows for monochromatic sources to be reconstructed at one given measurement due to the achromatic nature of the geometric phase. Hence, it can be used to measure monochromatic spectra or it may include multiple measurements at several wavelengths (e.g., Red/Green/Blue), as a wavelength selector (monochromator). Nevertheless, it is shown the method is able to record more than one wavelength in one measurement provided that a detector array is used and the sources are spatially separated. As compared with a dispersive approach, the S/N is improved by a higher throughput (Jacquinot advantage)^20,21^ but not by multiplexing (Fellgett advantage)^20–22^. Similar devices, working on the principle of Fourier-transform spectroscopy, may use the birefringence of Wollaston prisms^23^, a single-mirror interferometer^24^, or even a design incorporating stepped mirrors^25,26^. Ultimately, they all can be categorized as scanning and static^27,28^ Fourier-transform spectrometers. In the first approach the interferogram is recorded by scanning it with a single-pixel detector, while the second implies no moving parts and the interferogram is recorded in a single shot with a detector array. From this perspective, our design is a scanning Fourier-transform device in which the interferogram is scanned by manipulating the geometric phase in the polarizing interferometer. However, compared with the above designs, our approach is not able to produce translation of interferograms of wide band radiation sources. Yet, it is a robust and flexible new design in which the spectral resolution and wavelength span can be adjusted simply by changing the rotation range and increment of an optical phase retarder.

This paper introduces the theoretical basis of the method and describes the proposed experimental setup used to validate it. The intrinsic monochromaticity of the method is depicted by using a theoretical comparison between the dynamic and geometric phase approach, respectively. After calibrating (using a HeNe laser as radiation source) the interferometer in terms of rotation angle of the optical retarder, we perform experimental measurements of the interferogram and reconstruct the monochromatic spectra for three low coherence sources (i.e., red, green and blue). In order to vindicate the main drawback of the measuring method, we give three possible applications, two of them being able to acquire multiple wavelengths in a given measurement.

## Methods

### Fourier-transform spectroscopy method

For an incident monochromatic radiation with the wavenumber $\widetilde{\nu }$ and intensity ${I}_{0}\left(\widetilde{\nu }\right)$, the intensity of the interference pattern as a function of the *OPD* is given by:

1 $${I}_{0}\left(x\right)={I}_{0}\left(\widetilde{\nu }\right)\left[1+\mathrm{cos}\left(2\pi \widetilde{\nu }x\right)\right],$$

where $\widetilde{\nu }=1/\lambda$ is the wavenumber or spatial frequency, *x* is the displacement and $\lambda$ the wavelength. Changing *x* by translation of the interferometer mirror results in a “cosine” variation of the spatial frequency, $\widetilde{\nu }$. When polychromatic radiation source is considered, a superposition of such “cosine” terms is obtained and if we subtract the average intensity in the interferogram, we get:

2 $${I\left(x\right)}_{interf}=I\left(x\right)-\stackrel{-}{I\left(x\right)}={\int }_{0}^{\infty }I\left(\widetilde{\nu }\right)\mathrm{cos}\left(2\pi \widetilde{\nu }x\right)d\widetilde{\nu .}$$

The source distribution, $I\left(\widetilde{\nu }\right)$, can be recovered by inverse Fourier transformation, as:

3 $$I\left(\widetilde{\nu }\right)={\int }_{-\infty }^{\infty }{I\left(x\right)}_{\mathit{interf}}\mathrm{ cos}\left(2\pi \widetilde{\nu }x\right)dx.$$

Given the infinite nature of the mathematical (theoretical) Fourier-transform function an apodization window must be applied. This is because physical measurements have a finite number of data points, hence the interferogram must be taken in a finite interval. By applying, for example, the Hann apodization window^29^ we get:

4 $$w\left(x\right)=0.5\left[1+\mathrm{cos}\left(\frac{2 \pi x }{\Delta N}\right)\right]={\mathrm{cos}}^{2}\left(\frac{\pi x}{\Delta N}\right),$$

where *ΔN* is the number of samples or the scan range of the interferogram. Usually, the range $\left[-\Delta N/2,\Delta N/2\right]$ is considered and the apodized interferogram can be expressed as:

5 $${I\left(x\right)}_{Hann}={\int }_{-\Delta N/2}^{\Delta N/2}w\left(x\right)I\left(\widetilde{\nu }\right)\mathrm{cos}\left(2 \pi \widetilde{\nu } x\right)d\widetilde{\nu }.$$

Incorporating such a window result in ripples around a peak in the spectrum; the steeper the window the bigger the ripples. Even though these unwanted effects may be dealt with to some extent by interpolating the spectrum it is desirable to choose the apodization window according to the application.

### Polarizing phase-shifting interferometer

**Figure 1:**
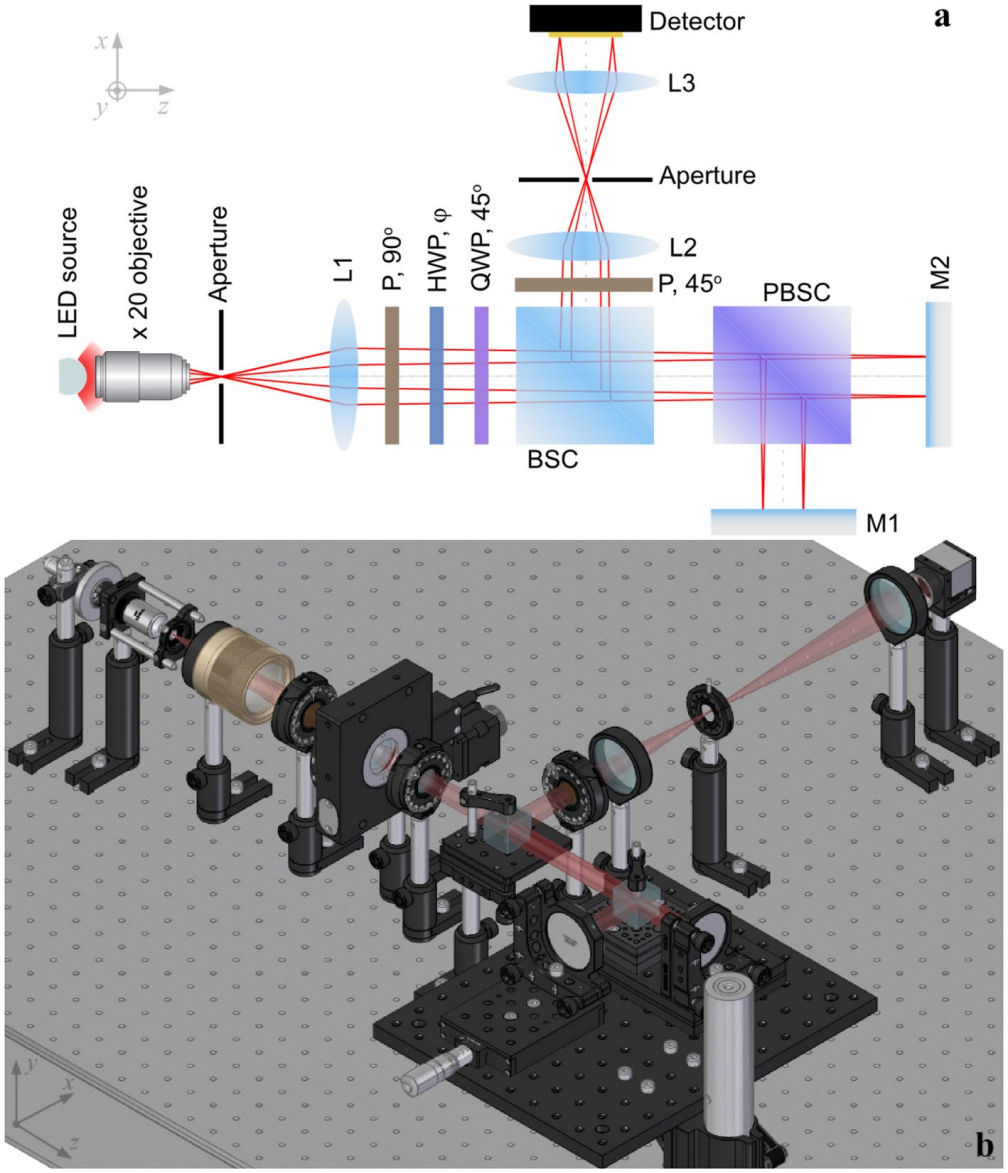
Experimental setup—(**a**) layout, (**b**) CAD design: light source, microscope objective, aperture, collimating lens (L1), linear polarizer aligned vertical to the optical table (P, 90°), half-wave plate with variable azimuth angle (HWP, φ), quarter-wave plate fixed azimuth angle at 45° (QWP, 45°), beam splitter cube (BSC), polarizing beam splitter cube (PBSC), mirrors (M1 and M2), linear polarizer at 45° (P, 45°), relay optics (L2 and L3) and detector.

In order to apply the Fourier-transform spectroscopy method using the geometric phase, a polarizing Twyman-Green interferometer (Fig. 1) working in low coherence light was designed. The low-coherence light sources are high-brightness light emitting diodes (LEDs)^30^. Light from these LEDs is focused on an aperture and then collimated with a photo lens (L1, *f* = 50 mm). The polarizing phase-shifter encompasses a HWP and a quarter-wave plate (QWP), both achromatic for the 460–680 nm wavelength range^31^. The QWP has the fast axis fixed at 45° with respect to the vertical axis, while the HWP is able to rotate around the optical axis. A linear polarizer (P, 90°) makes the incident light vertically polarized. Next, the beam passes through the rotating half-wave plate (HWP, φ), fixed quarter-wave plate (QWP, 45°), beam splitter cube (BSC) and polarizing beam splitter cube (PBSC); where *p*-polarized light is transmitted toward mirror M2 and *s*-polarized light is reflected toward mirror M1. The two beams are reflected on the mirrors and directed toward the detection arm via the BSC. A linear polarizer (P, 45°) brings the two beams to the same polarization in order for them to interfere. The two achromatic lenses, L2 and L3, are used to adjust the size of the interference pattern on the detector. With this configuration, the two polarization states *s* (i.e., vertical) and *p* (i.e., horizontal) are describing a closed path on the Poincare sphere. Hence, the phase of the radiation beam exiting the interferometer will be different from the one entering, while the *OPD* will be unchanged. In order to set the interferometer to *ZPD*, a *white light* LED is used as source and one of the mirrors is placed on a micrometer translation stage. Then, the distance between this mirror and the PBSC is finely tuned until interference is achieved. This is a daunting task due to the broad bandwidth of *white light*, but once reached, it provides the best alignment of the interferometer. Thus, it is the rotating phase-shifter that alters the phase by changing the polarization state of the two interfering waves during a measurement. If we consider the rotation angle required to acquire one fringe count at the detector, $\varphi$, we can write:

6 $$\Delta rot=N \varphi \left(degrees\, of\, arc\right),$$

where $\Delta rot$ is the total rotation of the HWP and *N* is the number of interference fringes, counted at the detector. Usually, the interferogram of the radiation source in a non-polarizing interferometer is achieved by translating one of the mirrors and acquiring an *OPD*. When a dark/ bright fringe takes the place of a similar adjacent one, the acquired *OPD* is equal to one wavelength of the radiation traveling in the interferometer.

At this point, we propose an artifact by associating the rotation scan of the HWP with an equivalent optical path difference (${OPD}_{e}$):

7 $${OPD}_{e}=2 d=N \lambda \text{(}cm\text{)},$$

where *d* is the distance, the mirror would have travelled in a non-polarizing interferometer and $\lambda$ is the wavelength. We called it equivalent because in a polarizing interferometer there is no actual *OPD* and the interferometer is kept at *ZPD* for the whole duration of the measurement. From Eqs. (6) and (7) it follows that:

8 $$\Delta rot= \frac{{OPD}_{e}}{\lambda } \varphi \left(degrees\, of \,arc\right).$$

At the same time, the equivalent increment of displacement or data interval, ${\delta x}_{e}$, is just:

9 $$\delta {x}_{e}= \frac{{OPD}_{e}}{N{\text{pts}}-1} \left(cm\right),$$

where *N*pts is the number of data points in the measurement. Similarly, the increment of rotation of the HWP in the polarizing interferometer, $\delta rot$, has the following expression:

10 $$\delta rot= \frac{\Delta rot}{N{\text{pts}}-1} \left(degrees\, of\, arc\right).$$

In order to compute the output signal as a function of the rotation angle of the HWP, $\varphi$, it is convenient to apply the Jones matrix formalism^32,33^, given that polarized light is used. Hence, the interference pattern at the detector can be expressed in terms of the amplitudes of the two orthogonally polarized waves, *E*_*s*_ and *E*_*p*_, as:

11 $${E}_{output}={E}_{s}+{E}_{p}={J}_{L}\left(45^\circ \right) {J}_{BS} {J}_{PBS\left(s\right)} {J}_{M} {J}_{PBS\left(s\right)} {J}_{R}\left(\frac{\pi }{2},45^\circ \right) {J}_{R}\left(\pi ,\varphi \right) {J}_{V}+{J}_{L}\left(45^\circ \right) {J}_{BS} {J}_{PBS\left(p\right)} {J}_{M} {J}_{PBS\left(p\right)} {J}_{R}\left(\frac{\pi }{2},45^\circ \right) {J}_{R}\left(\pi ,\varphi \right) {J}_{V},$$

where: *J*_*V*_ is the vector of linearly polarized wave along the *y* axis (vertical); *J*_*R*_ (*π*,*φ*)—matrix of the HWP with its fast axis rotated by *φ* degrees; *J*_*R*_ (*π/2*,45°)—matrix of QWP with the fast axis at 45°; *J*_*PBS*(*s*)_ and *J*_*PBS*(*p*)_ —matrices of the polarizing beam splitter for the reflected and transmitted wave, respectively; *J*_*M*_—matrix describing reflection on the mirror; *J*_*BS*_—phase introduced by reflection on the beam splitter cube; and *J*_*L*_ (45°)—matrix of the linear polarizer rotated at 45°. Using the expressions of the Jones matrices and vectors for the involved optical components and after some algebraic computations, the total output electric field is:

$${E}_{output}\left(\varphi \right)=\frac{1}{2}\left(\begin{array}{cc}1& 1\\ 1& 1\end{array}\right)\frac{1}{\sqrt{2}}\left(\begin{array}{cc}1& 0\\ 0& 1\end{array}\right)\left(\begin{array}{cc}0& 0\\ 0& 1\end{array}\right)\left(\begin{array}{cc}1& 0\\ 0& -1\end{array}\right)\left(\begin{array}{cc}0& 0\\ 0& 1\end{array}\right)\cdot \left(\begin{array}{cc}1/\sqrt{2}& i/\sqrt{2}\\ i/\sqrt{2}& 1/\sqrt{2}\end{array}\right)\left(\begin{array}{cc}i \mathrm{cos}\left(2\varphi \right)& i \mathrm{sin}\left(2\varphi \right)\\ i \mathrm{sin}\left(2\varphi \right)& -i \mathrm{cos}\left(2\varphi \right)\end{array}\right)\left(\begin{array}{c}0\\ 1\end{array}\right)$$

$$+\frac{1}{2}\left(\begin{array}{cc}1& 1\\ 1& 1\end{array}\right)\frac{1}{\sqrt{2}}\left(\begin{array}{cc}1& 0\\ 0& 1\end{array}\right)\left(\begin{array}{cc}1& 0\\ 0& 0\end{array}\right)\left(\begin{array}{cc}1& 0\\ 0& -1\end{array}\right)\left(\begin{array}{cc}1& 0\\ 0& 0\end{array}\right)\cdot \left(\begin{array}{cc}1/\sqrt{2}& i/\sqrt{2}\\ i/\sqrt{2}& 1/\sqrt{2}\end{array}\right)\left(\begin{array}{cc}i \mathrm{cos}\left(2\varphi \right)& i \mathrm{sin}\left(2\varphi \right)\\ i \mathrm{sin}\left(2\varphi \right)& -i \mathrm{cos}\left(2\varphi \right)\end{array}\right)\left(\begin{array}{c}0\\ 1\end{array}\right)$$

$$=\frac{1}{4}\left(i \mathrm{cos}\left(2\varphi \right)+\mathrm{sin}\left(2\varphi \right)\right)\left(\begin{array}{c}1\\ 1\end{array}\right)+\frac{1}{4}\left( \mathrm{cos}\left(2\varphi \right)+i \mathrm{sin}\left(2\varphi \right)\right)\left(\begin{array}{c}1\\ 1\end{array}\right)$$

12 $$=\left(\frac{1}{4}+\frac{i}{4}\right)\left[\mathrm{cos}\left(2\varphi \right)+\mathrm{sin}\left(2\varphi \right)\right]\left(\begin{array}{c}1\\ 1\end{array}\right)= \frac{1}{4} {e}^{-2i\varphi } \left(i+ {e}^{4i\varphi }\right) \left(\begin{array}{c}1\\ 1\end{array}\right),$$

with the amplitudes of the two orthogonally polarized waves:

13 $${E}_{s}=\frac{1}{4} {e}^{i \left(\frac{\pi }{2} -2\varphi \right)}\left(\begin{array}{c}1\\ 1\end{array}\right) {\text{and}} {E}_{p}=\frac{1}{4} {e}^{2i\varphi }\left(\begin{array}{c}1\\ 1\end{array}\right).$$

From this result it is obvious the two waves exit the interferometer linearly polarized at 45°. Next, the measured intensity as a function of azimuth angle of the HWP is:

14 $$I\left(\varphi \right)={I}_{avg} {E}_{output}\cdot {E}_{output}^{*}={I}_{avg}\frac{1}{4} {e}^{2i\varphi }\left[-i+{e}^{-4i\varphi }\right] \left(\begin{array}{cc}1& 1\end{array}\right) \cdot \frac{1}{4} {e}^{-2i\varphi }\left[i+{e}^{4i\varphi }\right]\left(\begin{array}{c}1\\ 1\end{array}\right)=\frac{1}{4} {I}_{avg} \left[1+{\text{sin}}\left(4\varphi \right)\right],$$

where

15 $${E}_{output}^{*}=\frac{1}{4} {e}^{2i\varphi }\left[-i+{e}^{-4i\varphi }\right] \left(\begin{array}{cc}1& 1\end{array}\right),$$

is the complex conjugate of the total output electric field and ${I}_{avg}$ is the background intensity in the interference pattern.

## Results

### Monochromatic vs polychromatic spectrum reconstruction

**Figure 2:**
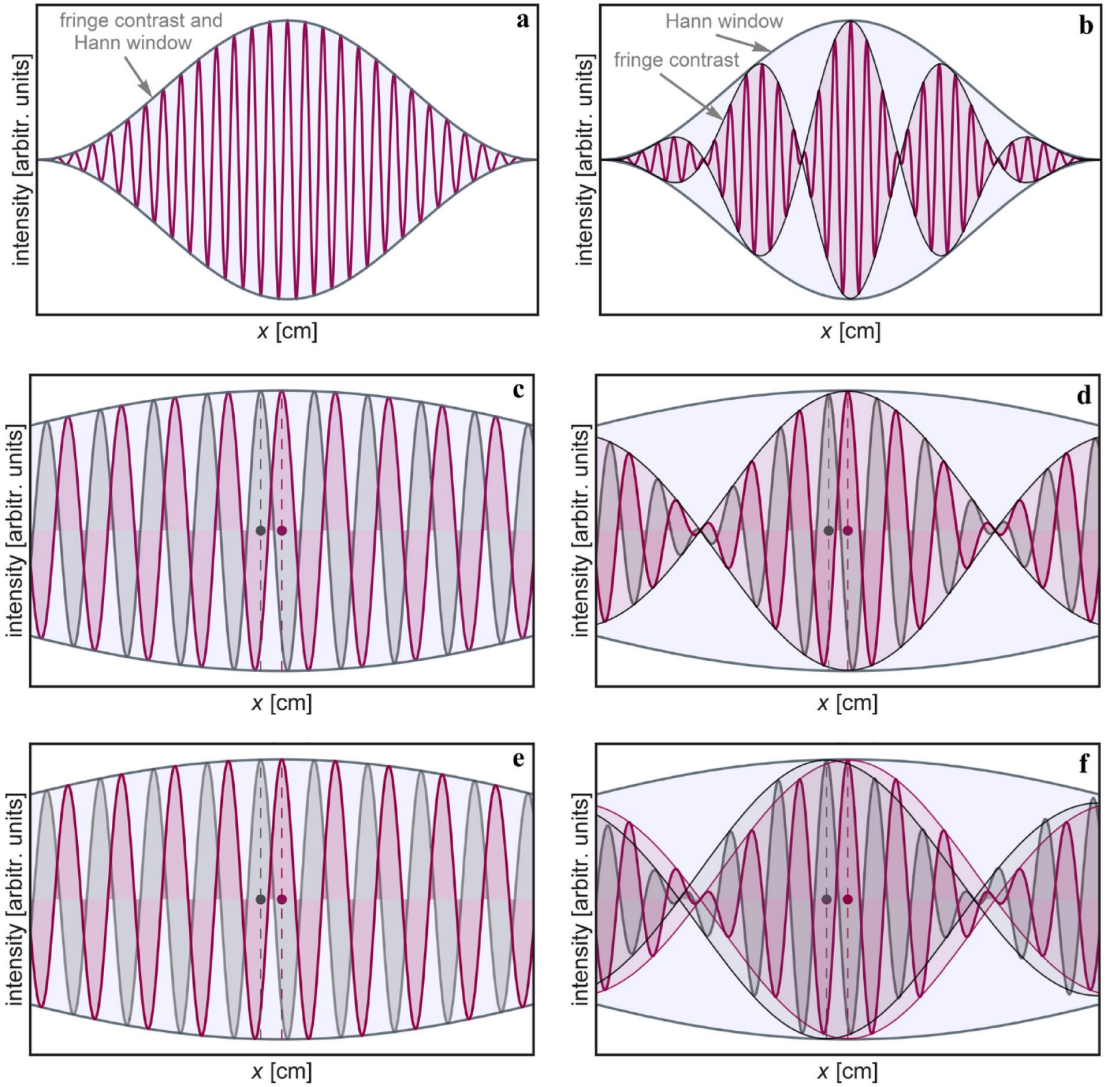
Theoretical simulation of a monochromatic (**a**) and polychromatic (**b**) apodized interferogram, respectively. The Hann apodization window envelope and interference fringes envelope (i.e., fringe contrast) are also depicted. The effect of the geometric phase shift on a monochromatic and polychromatic wave, respectively, is described in (**c**) and (**d**). Similarly, the effect of the dynamic phase shift on the same monochromatic and polychromatic waves is shown in (**e**) and, (**f**). In the monochromatic wave scenario, there are similar results for the two kinds of phase shift. In the case of a polychromatic wave, the geometric phase shift only modulates the interference fringes but with no translation of the fringe contrast envelope.

The scanning of the interferogram is obtained by implementing controlled phase shifts that alter the *OPD* and results in a translation motion/ scanning of the detected interference pattern. This is readily achieved in the case of dynamic phase, as it directly controls the *OPD* and is wavelength dependent. When geometric phase is used for the task, it turns out, only interferogram of monochromatic light can be scanned in order to reconstruct the spectrum. For comparison, both a monochromatic (Fig. 2a) and polychromatic (Fig. 2b) interferogram were simulated.

Next a phase shift was inserted for each case, such that to simulate the scanning of the interferogram in a Fourier-transform spectrometer. This phase shift is described by plotting two interferograms with a phase difference between them. For clarity, it was zoomed-in on the central part of these interferograms. In the case of the monochromatic wave, both the geometric (Fig. 2c) and dynamic (Fig. 2e) phase shift produces the same effect: a translation of the interferogram. For the polychromatic wave, the geometric phase shift (Fig. 2d) produces a modulation of the interference fringes while the dynamic phase shift (Fig. 2f) produces the translation of the interferogram. A scanning of the interferogram—which is needed in order to reconstruct the spectrum by Fourier transformation—occurs only when the envelope of the interference fringes (i.e., fringe contrast) makes a translation due to a phase shift. Figure 2c,e,f show such a behavior, while Fig. 2d depicts only a modulation of the interference fringes inside the fringe contrast envelope. Thus, due to the topological nature of the geometric phase, it can only acquire scanning of the interferogram for monochromatic waves.

### Calibration of the interferometer

**Figure 3:**
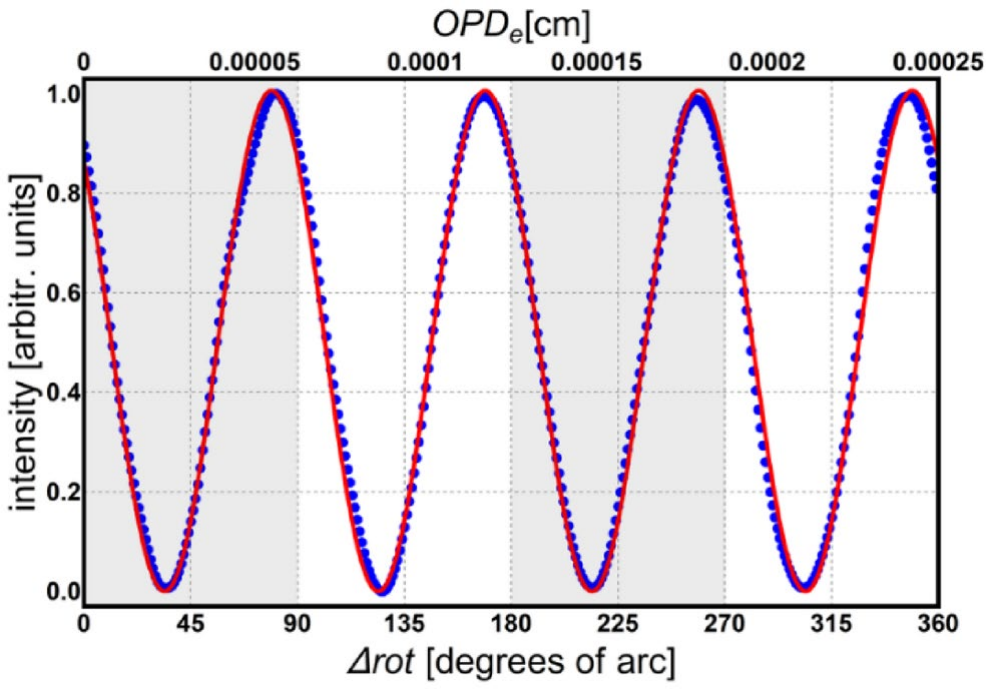
Calibration of the polarizing interferometer: blue dots—experimental data, red curve—theoretical model.

The phase difference between the two interfering waves changes with the azimuth angle of the HWP and only the geometric phase is altered. Thus, the polarizing interferometer must be calibrated in terms of the rotation angle and fringe displacement at the detector. For that, the interferogram produced by a stabilized HeNe laser (wavelength of 632.8 nm) was scanned for each 0.5° rotation increment of the HWP. An intensity vs rotation angle of the HWP graph is achieved, as shown in Fig. 3. Fitting this graph with an expression similar to the one in Eq. 1 (where the dynamic phase term, $2\pi \widetilde{\nu }x$, is replaced by the geometric phase term, $4\varphi$) results in a value of 90° for the rotation angle $\widehat{\varphi }$ (see Eq. 6) when one fringe is counted at the detector. An equivalent optical path difference of 2.5 μm is also obtained for the full 360° rotation of the HWP.

### Measurements and data processing

The measurements consist in recording a number of interference images corresponding to the number of data points, *N*pts, for each rotation increment of the HWP, $\delta rot$.

Then, the intensity value of the same pixel in each image is extracted and an interferogram, describing intensity as a function of the total rotation, $\Delta rot$, is achieved; this is similar to a scanning of the interferogram with a single-pixel detector. Starting with the values for the wavelength of the calibration laser (*λ*_HeNe_ = 632.83 nm), the number of data points, *N*pts, and knowing that:

11 $$\Delta rot= \delta rot \left(Npts-1\right),$$

we give values to $\delta rot$ such that to obtain an integer number of fringes, *N*, counted at the detector:

12 $$N=\frac{\Delta rot}{\varphi }=\frac{\Delta rot}{90^\circ }.$$

**Figure 4:**
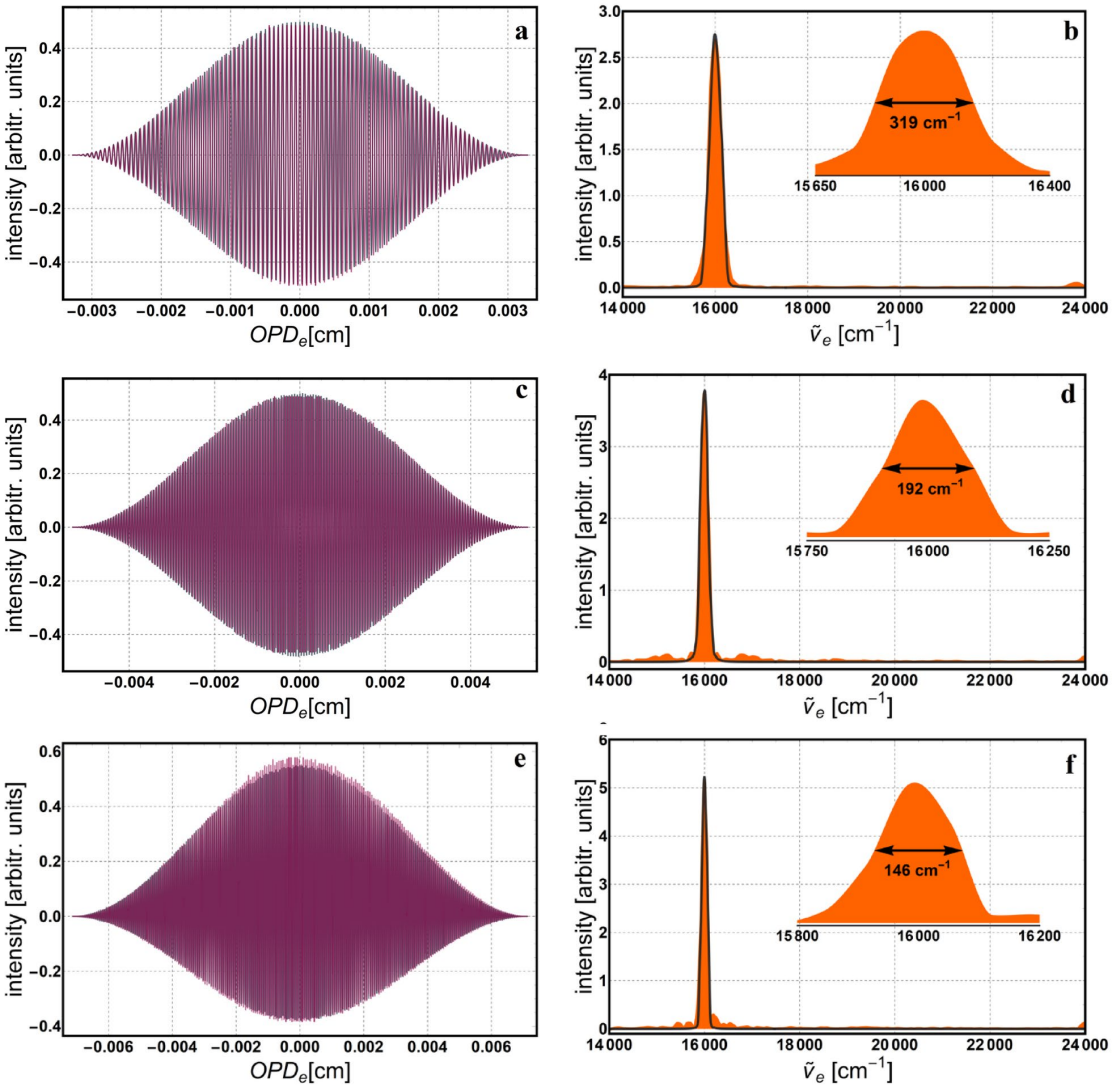
Simulation (gray) and experimental (color) reconstruction of both, apodized interferogram and the corresponding monochromatic spectrum for the 625 nm source: (**a**,**b**) 512; (**c**,**d**) 1024 and (**e**,**f**) 2048 data points. Insets show the enlarged area around the peak, with the corresponding spectral width value computed as the FWHM.

**Figure 5:**
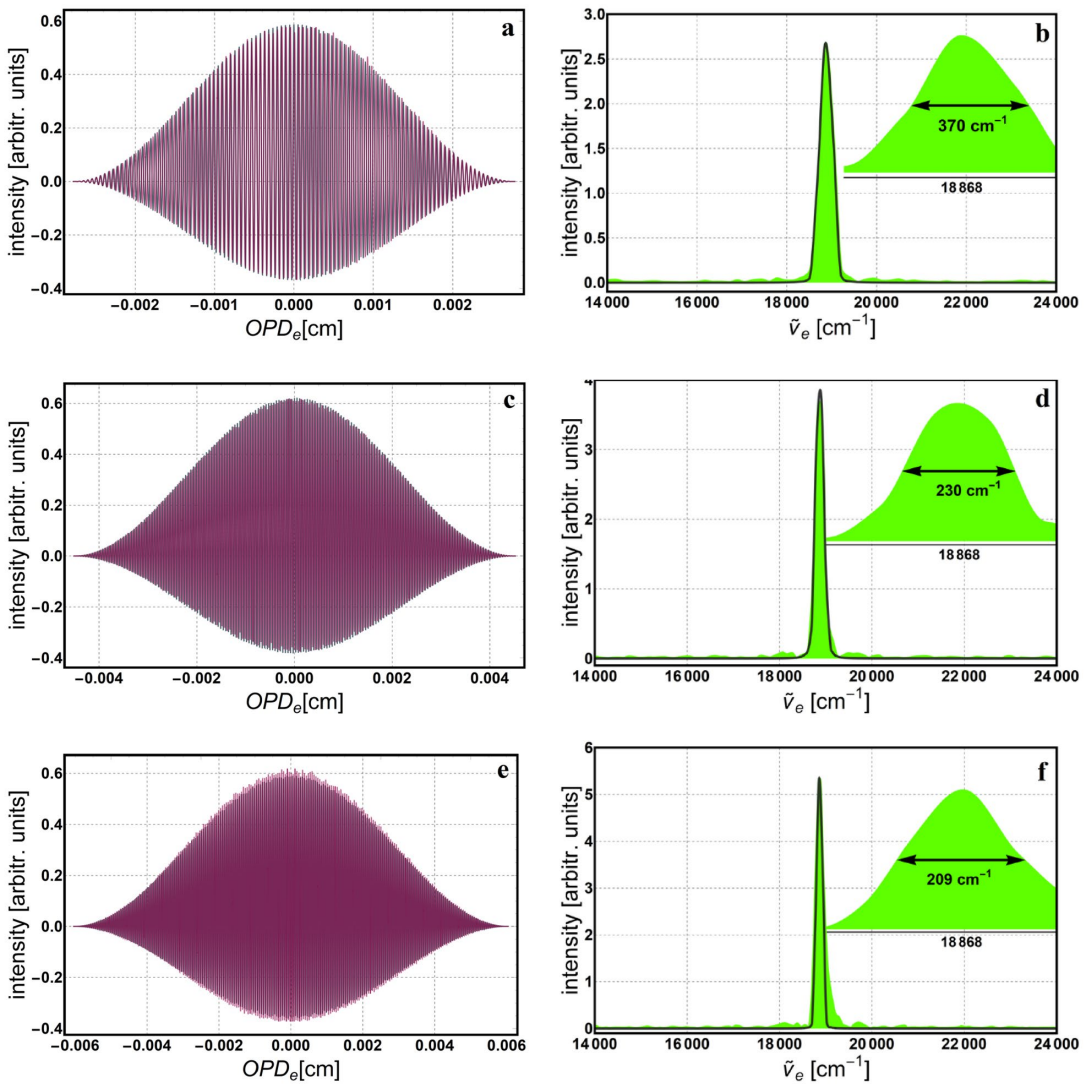
Simulation (gray) and experimental (color) reconstruction of both apodized interferogram and the corresponding monochromatic spectrum for the 530 nm source: (**a**,**b**) 512; (**c**,**d**) 1024 and (**e**,**f**) 2048 data points. Insets show the enlarged area around the peak, with the corresponding spectral width value computed as the FWHM.

**Figure 6:**
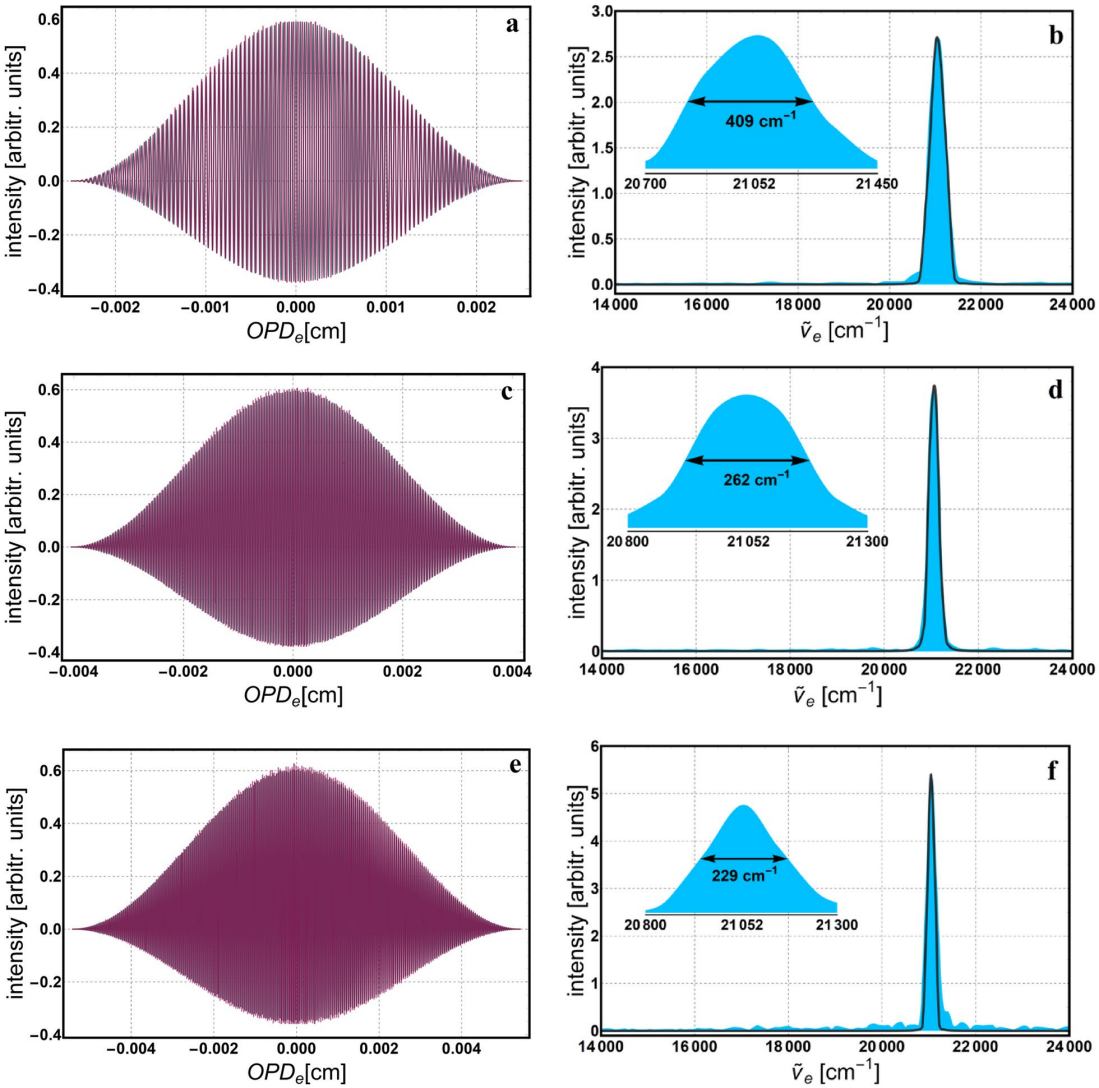
Simulation (gray) and experimental (color) reconstruction of both apodized interferogram and the corresponding monochromatic spectrum for the 475 nm source: (**a**,**b**) 512; (**c**,**d**) 1024 and (**e**,**f**) 2048 data points. Insets show the enlarged area around the peak, with the corresponding spectral width value computed as the FWHM.

In return, we are able to compute the ${OPD}_{e}$, resolution ($Res=1/{OPD}_{e}$), and the rest of the parameters defined in Section *Polarizing phase-shifting interferometer*, as depicted in Table 1. While adjusting the number of fringes we look to achieve reasonable values for the rotation increment and spectral resolution. The number of fringes, *N*, is intentionally chosen to be an integer number because, together with an appropriate windowing (apodization) function, it helps reducing the inherent *spectral leakage*^34^. This is because a whole number of periods in the interferogram facilitates a correct Fourier transformation and consequently, a true distribution of the spectrum. Next, the interferogram describing variation of intensity as a function of the ${OPD}_{e}$ is reconstructed, and then, the apodization window is applied. Finally, the Fourier transformation is applied to reconstruct the monochromatic spectrum. Figures 4, 5 and 6 show the interferogram and its corresponding spectrum for 512, 1024 and 2048 data points. Both, the interferogram and spectrum are simulated (gray) and experimental (color). These measurements are realized with three LED sources having the following central wavelengths: 625 nm—red LED, 530 nm—green LED and 475 nm—blue LED, respectively. Insets in Figs. 4, 5 and 6 show spectral linewidth values, computed as the Full Width at Half Maximum (FWHM). The linewidth gets thinner with better resolution, which improves with increasing $\Delta rot$ (or ${OPD}_{e}$), and consequently with the number of data points. Also, the spectra data were interpolated using the *Akima* spline^35^ to smooth it and reduce ripples due to the apodization window. Because we worked in the space-domain all spectra are depicted in equivalent wavenumbers (${\widetilde{\nu }}_{e}$) and the corresponding values for the illumination sources above are: 16,000 cm^−1^—red LED, 18,867 cm^−1^—green LED and 21,052 cm^−1^—blue LED.

**Table 1:** Measurement parameters for several spectral resolution settings.

*N*pts	*Res* (cm^−1^)	${OPD}_{e}$ (cm)	$\delta rot$ (°)	*N*	$\Delta rot$ (°)	$\delta {x}_{e}$ (nm)
512	150.4	0.00664	18.5	105	9453.5	130
1024	92.4	0.01082	15	171	15,390	105.8
2048	69.3	0.01442	10	228	20,520	70.5
4096	38.5	0.02595	9	410	36,900	63.4
8192	28.9	0.03461	6	547	49,230	42.3
16,384	17.3	0.05765	5	911	81,990	35.2

*Npts* number of data points, *Res* spectral resolution, ${OPD}_{e}$ equivalent *OPD*, $\delta rot$ rotation increment of the HWP, *N* number of counted fringes, $\Delta rot$ accumulated rotation, $\delta {x}_{e}$ equivalent displacement increment.

**Figure 7:**
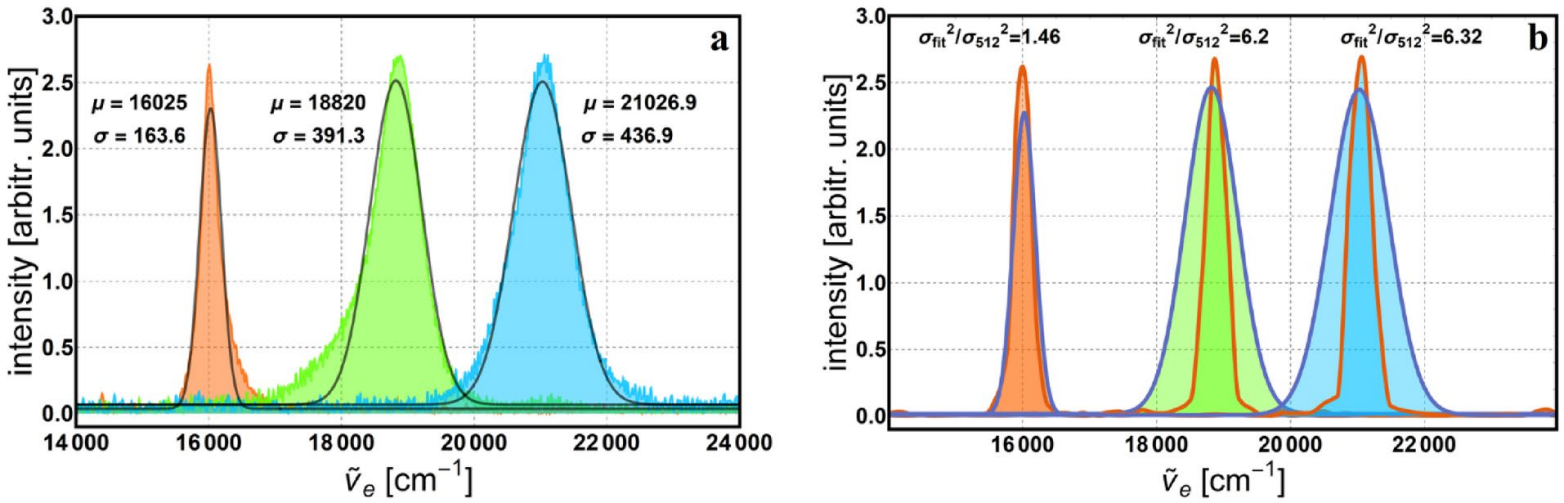
Composition of the individually measured spectra for: (**a**) ARCspectro HT-HR commercial spectrometer with the corresponding Gaussian fittings (gray line), (**b**) geometric phase method for 512 data points (orange line) and the Gaussian fittings of the commercial spectrometer (blue-indigo line).

Notice, there are only two interferograms (Fig. 4a,c) that are symmetric around the average value, y = 0. Usually, if there are no losses within the optical components or stray reflections, they all should be symmetric. In our case, the anomaly is due to the sensitivity of the detector, which can be approximated by the sigmoid logistic function. Hence, for the two symmetric cases, the intensity minimum and maximum values in the interference image, were situated on the linear middle part of this sensitivity curve. For the rest of the interference images, where the overall intensity was lower, it seems the minimum is situated on the lower part of the curve, where the gain in voltage is not linear but slightly higher; hence the asymmetry around the mean value. The composition of individually measured spectra of the same light sources but taken with a commercial spectrometer (ARCspectro HT-HR)^36^ is depicted in Fig. 7a, together with their corresponding Gaussian fittings, $y=a\mathrm{exp}\left(-{\left(x-\mu \right)}^{2}/2{\sigma }^{2}\right)+c$, where *a* and *c* are constants, *μ* is the mean value and *σ* is the standard deviation. Following these fittings, the mean and standard deviation get the next values: ${\mu }_{R}=16025\, {\text{cm}}^{-1}$, ${\mu }_{G}=18820 \,{\text{cm}}^{-1}$, ${\mu }_{B}=21026.9 \,{\text{cm}}^{-1}$, ${\sigma }_{R}=163.6$ cm^-1^, ${\sigma }_{G}=391.3$ cm^-1^ and ${\sigma }_{B}=436.9$ cm^-1^. For comparison, the spectra for 512 data points and Gaussian fittings of the spectra recorded with the commercial spectrometer are shown in Fig. 7b. Notice the mean value of each peak varies slowly from the central value (i.e., 16,000 cm^−1^—red LED, 18,867 cm^−1^—green LED and 21,052 cm^−1^—blue LED) and the standard deviation is larger, especially for the blue and green LEDs. In order to quantize the difference between these spectra, we used the ratio of the corresponding variances and obtained the following values: $(\sigma_{(fit (R))^2})/(\sigma_{(512 (R))^2 })=1.46$, $(\sigma_{(fit (G))^2})/(\sigma_{(512 (G))^2 })=6.2$, and $(\sigma_{(fit (B))^2})/(\sigma_{(512 (B))^2 })=6.32$ respectively. To compute the variances of the 512 data points spectra, the relation ${\text{FWHM}}=2\sqrt{2 {\text{ln}}2} \sigma$ was used. Even though these monochromatic spectra follow the rule of increasing resolution with the number of data points, it is important to keep in mind that our method is exclusively monochromatic. Therefore, the contributions to the spectrum from neighboring wavelengths are not considered and the linewidth appears thinner than it actually is for a given resolution. As mentioned above, the monochromaticity of the geometric phase limits the method to wavelength recognition and not genuine spectral reconstruction. Nonetheless, we identified possible applications and gave a brief description below. One of the applications is to use the method as a wavelength selector. For instance, we placed a green filter (532 nm central wavelength) in the path of radiation from a white LED, in order to select and detect only the specified wavelength.

**Figure 8:**
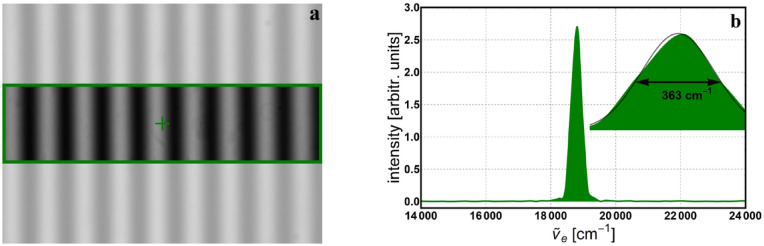
White LED light through a green filter with central wavelength of 532 nm: (**a**) interferogram with selected area and position of the pixel used for processing; (**b**) reconstructed monochromatic spectrum for 512 data points and its Gaussian fit.

Using a detector array (CCD camera) in a single-pixel detection scheme can give some advantage. The second application was to use a green LED (530 nm central wavelength) to illuminate the interferometer and use the green filter to cover only the top half of the interferogram recorded at the CCD. Figure 8a shows one of the interference images, the selected area for processing and position of the pixel used in the image processing. Figure 8b depicts the achieved monochromatic spectrum of the selected wavelength from the *white light* source.

**Figure 9:**
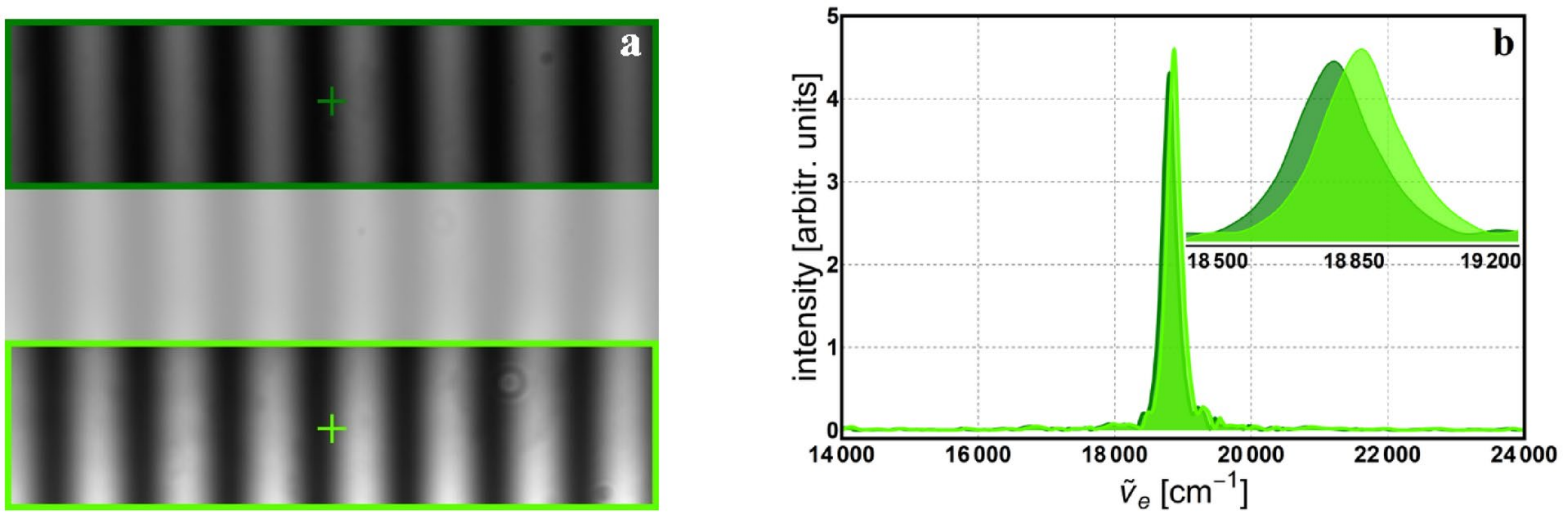
Green LED light (530 nm) with top half passed through a green filter (532 nm): (**a**) interferogram with selected areas and positions of the pixels used in the processing; (**b**) reconstructed monochromatic spectra for 2048 data points.

This way, we still take a single measurement (scanning), but the image processing is done separately for the top half and bottom half of the images, as shown in Fig. 9a. This is also allowed because of the achromatic nature of the geometric phase, which is used to control the scanning. So, by using a CCD detector, two (or more) monochromatic spectra (Fig. 9b) can be recovered by a single measurement, but processing must be carried out separately for the corresponding area in the interferogram.

**Figure 10:**
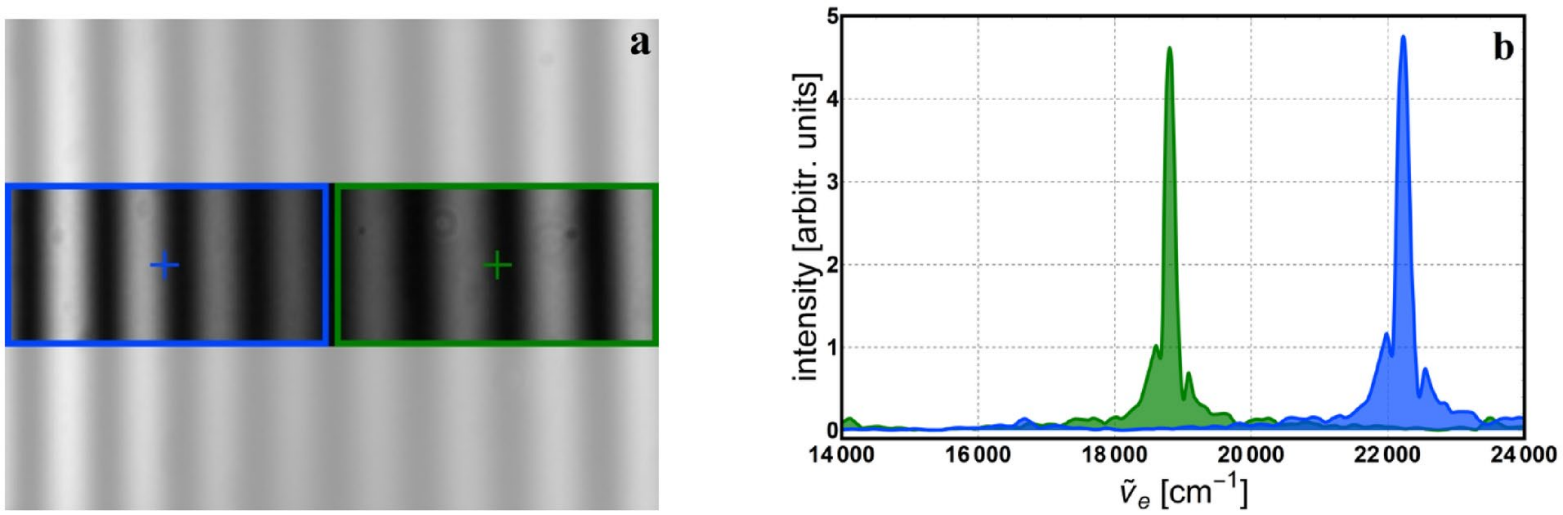
White LED light with left half passed through a blue filter (450 nm) and right half through a green filter (532 nm): (**a**) interferogram with selected areas and positions of the pixels used in the processing; (**b**) reconstructed monochromatic spectra.

In the third application, a white LED is used to illuminate the interferometer while two filters (blue @ 450 nm and green @ 532 nm central wavelengths) are positioned to cover the left and right part of the image, respectively. Again, only a single measurement is required but separate processing of the various parts in the interferogram need to be carried out, as shown in Fig. 10a. The corresponding monochromatic spectra are depicted in Fig. 10b.

## Conclusions

The paper shows that geometric phase in a polarizing interferometer can be used as a phase shifter mechanism for light measurement using Fourier-transform spectroscopy. In order to assess this method both theoretical and experimental work was carried out. It was found that only monochromatic radiation can be measured due to the achromatic nature of the geometric phase. Even though the method does not allow full spectroscopic measurements, a couple of possible applications were identified and described. It is possible to measure multiple wavelengths at once by assuring spatially separated sources and a CCD (not a single-pixel detector) as detector. We have chosen LEDs as light sources to illustrate the technique works well in low coherence light but it can be applied to any radiation source, once the interferometer is aligned to zero path difference. The main advantage of the geometric phase approach over the dynamic phase is that we get to control rotations of degrees of arc instead of translations on the order of tens of nanometers for measurements on the same spectral range (visible range, in this case). Thus, not only translation is replaced with rotation motion of the phase shifter, the amount of this motion is considerably larger and, in consequence, easier to control with less noise involved.
